# High Anti-CoV2S Antibody Levels at Hospitalization Are Associated with Improved Survival in Patients with COVID-19 Vaccine Breakthrough Infection

**DOI:** 10.3390/ijerph192315581

**Published:** 2022-11-24

**Authors:** Matthias Wolfgang Heinzl, Lisa Kolenchery, Michael Resl, Carmen Klammer, Anne Black, Florian Obendorf, Lukas Schinagl, Roland Feldbauer, Johannes Pohlhammer, Thomas Wagner, Thomas Berger, Benjamin Dieplinger, Martin Clodi

**Affiliations:** 1 Department of Internal Medicine, Konventhospital Barmherzige Brueder Linz (St. John of God Hospital Linz), 4020 Linz, Austria; 2 ICMR—Institute for Cardiovascular and Metabolic Research, Johannes Kepler Universität Linz (JKU Linz), 4020 Linz, Austria; 3 Department of Laboratory Medicine, Konventhospital Barmherzige Brueder Linz (St. John of God Hospital Linz), 4020 Linz, Austria

**Keywords:** COVID-19, Delta variant, anti-CoV2S antibody, vaccine breakthrough infection, outcome prediction, mortality

## Abstract

Background: Although vaccination against COVID-19 is highly effective, breakthrough infections occur, often leading to severe courses and death. The extent of protection provided by individual antibody levels in breakthrough infections is still unknown and cut-off levels have yet to be determined. Methods: In 80 consecutive fully vaccinated patients hospitalized between August and December 2021 with COVID-19 breakthrough infection (Delta variant), anti-CoV2S antibody levels were analyzed for the endpoint of death. Results: Ten out of the 12 patients who died (83.3%) had antibody levels < 600 U/mL; 5 (41.7%) of these had antibody levels < 200 U/mL. Only 2 patients with a level of >600 U/mL died from vaccine breakthrough infection. Correction for the number of comorbidities and age revealed that anti-CoV2S antibody levels at the time of hospitalization were a significant predictor for reduced risk of death (OR = 0.402 for every 1000 U/mL, *p* = 0.018). Conclusions: In this retrospective data analysis, we show that almost all patients who died from COVID-19 vaccine breakthrough infection had antibody levels < 600 U/mL, most of them below 200 U/mL. In logistic regression corrected for the number of comorbidities and age, anti-CoV2S antibody levels at the time of hospitalization proved to be a significantly protective predictor against death.

## 1. Introduction

COVID-19 is an infection caused by SARS-CoV-2 that mainly affects the upper respiratory tract and lungs. The virus contains two main proteins: nucleocapsid (N) and spike (S) protein. The spike protein is important for the interaction with host cells that express the Angiotensin Converting Enzyme 2 (ACE-2) receptor [1]. The first case of COVID-19 was reported in Wuhan, Hubei (China) in December 2019 [2].

Patients with comorbidities have a higher risk for severe disease than patients without pre-existing conditions. The comorbidities of diabetes, high blood pressure, cardiovascular diseases and COPD are associated with 2.3-fold increased mortality [3]. In terms of symptoms, fever and cough are the most common symptoms of COVID-19. Furthermore, fatigue and dyspnea are generally very common in patients with SARS-CoV-2 infection [4]. Dyspnea is significantly associated with a severe infection and the need for intensive care unit (ICU) treatment [3].

The most commonly administered vaccines in Austria and central Europe are Janssen Ad26.COV2.S, Moderna mRNA-1273, BionTech Pfizer BNT162b2 and AstraZeneca ChAdOx1 [5]. Vaccine efficacy against hospitalization and mortality is given as 42–79% for BNT162b2, 47–79% for Janssen vaccine, 60–67% for ChAdOx1 and 64% for Moderna [6].

In the literature, cases of breakthrough infections have been described. They are defined as SARS-CoV-2 infection with a positive PCR test in people who are considered fully vaccinated according to the vaccine-specific schemes at the time, i.e., the single vaccination for Janssen Ad26.COV2.S and the double vaccination for the other vaccines at least 14 days previously [7]. Several studies showed that the anti-CoV2S antibody level has a positive correlation with protection against SARS-CoV-2 infection [8,9,10]. 

Since the start of the pandemic, several variants have been detected, with different specific properties. During the course of 2022, sublineages of the Omicron variant have caused the vast majority of COVID cases worldwide as well as in Austria. Before the Omicron variant, the Delta variant caused an important wave of infections, including many breakthrough infections and often leading to hospitalization. In contrast to previous variants, the neutralizing effects of antibodies are significantly reduced in the Delta variant. Several reasons including mutations in the spike protein of the virus are discussed, leading to more severe infections with the Delta variant [11]. 

Aldridge et al. showed, that an anti-CoV2S level ≥ 500 U/mL has a reduced risk of breakthrough infections after the second vaccination dose [7]. However, it is not clearly described in the literature which antibody level protects against severe infection or death in fully vaccinated people with subsequent SARS-CoV-2 infection with the Delta variant and whether high levels of antibodies following vaccination are predictive of an improved outcome.

Therefore, we examined 80 fully vaccinated patients with SARS-CoV-2 vaccine breakthrough infection to analyze their antibody level. The aim of this retrospective data analysis was to determine which patients have a higher risk of death. The effect of SARS-CoV-2 infection on the antibody kinetics in vaccinated persons is also investigated.

## 2. Methods

This study is a retrospective data analysis of 80 patients hospitalized due to breakthrough infection with the Delta variant of SARS-CoV-2 despite full basic immunization according to the vaccination-recommendations at the time. The data collection took place in the Department of Internal Medicine at Saint John of God Hospital Linz (Austria) from August to December 2021. 

Patients had received at least 2 vaccine doses of AstraZeneca, BionTech Pfizer, Moderna or 1 dose of Janssen vaccine according to the guidelines at the time. The data were collected from the electronic patient file using the following documents: electronic patient file with doctor’s letters, work and out-patient documentation. Data on symptoms, personal information, vaccination, treatment, complications, and comorbidities were collected through medical history. 

We only included patient data with a positive polymerase chain reaction (PCR) test for SARS-CoV-2. The virus was detected in all patients on the first day of hospitalization using a PCR test from nasopharyngeal swabs. Confirmation of the Delta variant was performed using real-time PCR (LightCycler^®^ cobas z 480 Analyzer, Roche, Mannheim, Germany, with TIB MOLBIOL VirSNiP SARS-CoV-2 Spike Assays).

The anti-SARS-CoV-2 spike IgG antibody (anti-CoV2S) levels were measured on the day of presentation, as well as during hospital stay, using a CE-IVD-marked quantitative immunoassay (Elecsys Anti-SARS-CoV-2S; Roche, Mannheim). The measuring range was between 0.41 and 2500 U/mL. The assay was calibrated with the manufacturer’s standard according to the First WHO International Standard for Anti-SARS-CoV 2-Immunglobulin (human), NIBSC-Code: 20/136 (Pearson correlation coefficient r = 0.9996). Therefore, the numeric value in U/mL is equivalent to the numeric value in the internationally standardized Binding Antibody Units (BAU)/mL. 

The patient data were anonymized to ensure data protection. The retrospective data analysis was approved by the local ethics committee of the medical faculty of JKU Linz (11 May 2022; 1073/2022).

### Statistics and Data Analysis 

Patient data were evaluated in the calculation program using descriptive statistics. We calculated the arithmetic mean, minimum, maximum and standard deviation for continuous variables and used frequency and percentages for categorial variables. A Mann–Whitney-U test was used for non-normally distributed variables to calculate the significance of the mean antibody level difference of the deceased and the survivors. For statistical analysis *p* values < 0.05 were considered statistically significant. To interpret antibody levels, we defined subsets and used level intervals in increments of 200 U/mL. The subset with level > 2400 includes all samples detected above the upper measuring limit of the laboratory. Thus, we identified how many patients were included in the respective subgroups to determine the association between antibody level and ICU or mortality. To present the results graphically, we used bar charts and a boxplot chart. Furthermore, a logistic regression model was calculated to determine the possible predictive value of patients’ age, the number of comorbidities (increased BMI > 25; malignant disease, pulmonary disease such as COPD or pulmonary fibrosis, cardiovascular disease, immunosuppression, arterial hypertension and diabetes mellitus) and antibody titer. For this calculation, values below the threshold of detection (<0.41 U/mL) were set to 0 U/mL and values above the detection range (>2500 U/mL) were set to 2500 U/mL.

## 3. Results

### 3.1. Patient Characteristics 

From August to December 2021 80 fully vaccinated patients with vaccination breakthrough infection were hospitalized, including 5 women (6.25%) and 75 men (93.75%). All patients had their second vaccination > 14 days ago. The mean age was 68.98 years (SD 15.16). The youngest patient was an 18-year-old man, and the oldest patient was a 97-year-old woman. Of note, due to structural peculiarities in the hospital system in Linz, most patients admitted to our institution are male, with the majority of female patients being admitted to another hospital.

In 72 patients (90%), we were able to determine the onset of symptoms from medical history. The mean duration of symptoms to hospitalization was 5.3 days (SD 4.31). The maximum was 21 days, and the minimal duration was 1 day, meaning the symptom onset was on the same day. In four patients (5%), we could not determine the exact time of onset due to lack of communication with the patients. In addition, four patients (5%) could not name the exact duration of their symptoms and named “several” days.

Most patients reported fever (50%) and dyspnea (48.75%) as the main symptoms on the day of the hospitalization. Forty-three patients (53.75%) had either a dry cough (41.25%) or a productive cough (12.5%). All patients were included during the Delta wave and infected with the Delta variant of SARS-CoV-2. This was additionally confirmed by real-time PCR testing in 44 of the 80 patients.

Sixty-one patients (76.25%) were immunized with at least 2 doses of BionTech Pfizer vaccine, while 4 out of 61 patients (6.55%) received three doses of BionTech Pfizer vaccine. Nine patients (11.25%) received AstraZeneca vaccine, 5 (6.25%) Janssen vaccine and 4 (5%) Moderna vaccine. Only one person (1.25%) was cross-vaccinated with two doses of AstraZeneca and 1 dose of BionTech Pfizer. Three out of 9 patients (33.3%) who received AstraZeneca vaccine died after COVID-19 breakthrough infection. In comparison to that, 8 out of 61 patients (13.11%) vaccinated with BionTech Pfizer died.

Regarding comorbidities, we found 43 patients (53.75%) with cardiovascular disease, 45 (56.25%) with arterial hypertension and 33 (41.25%) with elevated body mass index (>25 kg/m^2^). The mean number of comorbidities was 2.3. 

On average, the patients stayed in hospital for about 10 days (SD 8.01). Thirteen patients of the entire cohort (16.25%) had to be treated in the intensive care unit (ICU) with a mean stay of 11.08 days (SD 6.28), while 11 out of these 13 (84.6%) were endotracheally intubated. Thirty-five patients (43.75%) were treated with noninvasive ventilation or high-flow oxygen. The most common complications that occurred due to COVID-19 were pneumonia (n = 49; 61.25%), respiratory insufficiency (n = 32; 40%) and bacterial superinfection (n = 21; 26.25%). Thirteen patients (16.25%) died, 6 of them in the ICU. One out of 13 of the deceased patients had to be excluded from the subgroup analysis due to lack of anti-CoV2S antibody measurements on the day of hospitalization (Table 1).

### 3.2. Anti-CoV2S Antibody Level 

In 76 patients (95%), anti-CoV2S antibody levels were measured on the day of consultation. Thirty-four patients (44.7%) had an anti-CoV2S antibody level of >2400 U/mL. 51.3% (n = 39) had a level of >1000 U/mL, 55.3% (n = 42) >800 U/mL and 57.9% (n = 44) >600 U/mL. 42.1% (n = 32) had a level < 600 U/mL.

**Figure 1:**
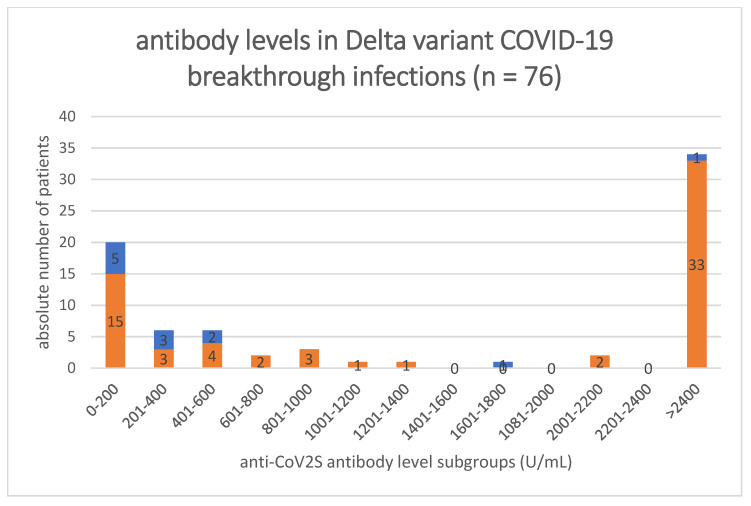
Subgroupanalysis of the anti-CoV2S antibody level in U/mL. Antibody levels were available in 76 of the 80 patients in this cohort. The anti-CoV2S antibody level is depicted in a range of 200 U/mL. The blue bars show the absolute number of deceased patients (n = 12). The orange bars show the absolute number of all patients with the respective level subgroups who survived (n = 64).

Twenty patients (26.3%) had a level between 0 and 200 U/mL, 6 (7.9%) between 201 and 400 U/mL and 6 (7.89%) between 401 and 600 U/mL (Figure 1). Of the 42 patients with an antibody level between 0 and 2400 U/mL 76.2% were <600 U/mL.

Of the patients with COVID-19 who died, 83.33% (n = 10) had an anti-CoV2S antibody level of <600 U/mL and only two patients (16.7%) who died had a level > 600 U/mL, namely, 1691 U/mL and >2400 U/mL. These two patients had multiple comorbidities and were of old age (85 or 90 years) (Figure 1). 

When comparing the anti-CoV2S levels in the different subgroups of hospitalized patients with COVID-19, we found three main results. First, most patients who died from COVID-19 had an antibody level < 600 U/mL. Five of these (41.7% of all deceased patients) had an anti-CoV2S antibody level of <200 U/mL. In the following group (201–400 U/mL) there was the second highest absolute number of patients (n = 3; 25%) who died from the infection. In contrast, only one person died with an anti-CoV2S antibody level > 2400 (n = 1; 8.3%). Similarly, regarding antibody levels in patients in need for ICU treatment), 6 of the 11 ICU patients had an anti-CoV2S antibody level < 600 U/mL.

We saw an important difference between patients with an antibody level < 600 U/mL and above, since 83.3% (n = 10) of the deceased had an anti-CoV2S antibody level below this. The patient who died despite the high level of > 2400 U/mL had multiple comorbidities, including epilepsy, hypertension and atypic Parkinson´s disease. The second person who died with a high level of >600, namely 1691 U/mL, also had Parkinson´s disease, arterial hypertension, coronary artery disease, atrial fibrillation and an advanced age of 90 years. 

**Figure 2:**
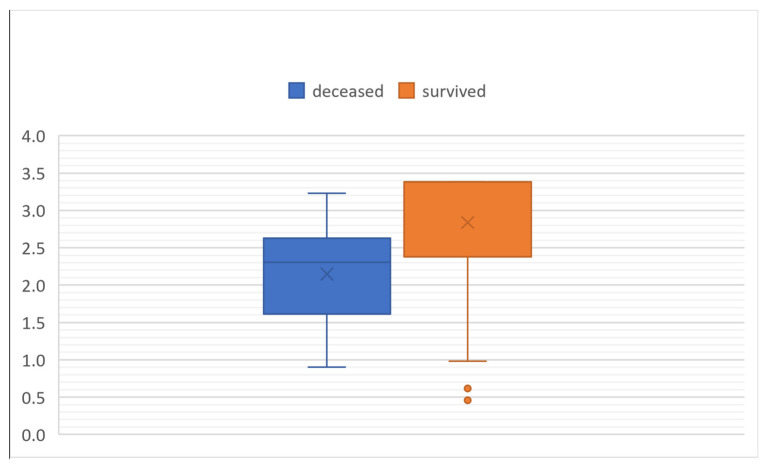
Comparison of the anti-CoV2S antibody level of the deceased and the surviving patients (n = 76). The mean level of antibodies at the time of hospitalization was lower in deceased patients (339.1 U/mL, blue) than it was in patients who survived (1481.8 U/mL, orange; *p* = 0.00152). For better visualization, the anti-CoV2S antibody level (U/mL) was converted to a decadic logarithmic scale. The blue boxplot demonstrates the level of the deceased patients and the orange one the level of the patients who survived.

The comparison of the mean anti-CoV2S antibody level of the deceased and the surviving patients showed significantly higher levels among surviving patients (mean level of antibodies: 339.1 U/mL vs. 1481.8 U/mL; *p* = 0.00152, see Figure 2).

### 3.3. Logistic Regression

Calculation of the logistic regression model including age, the number of comorbidities and anti-CoV2S antibody levels revealed that neither the number of comorbidities (OR 1.4, *p* = 0.284) nor patients’ age (OR 1.03, *p* = 0.282) were significant predictors for death. Anti-CoV2S antibody levels proved to be significantly negatively associated with death (*p* = 0.018, OR 0.402 for every 1000 U/mL).

## 4. Discussion

Vaccines against COVID-19 are protective against symptomatic disease. Nevertheless, breakthrough infections in fully vaccinated individuals do occur very frequently, especially since the Delta variant and Omicron variants have been circulating [12]. The level of anti-CoV2S antibodies following vaccination is correlated with neutralization potency [13] and is an important marker of protection against COVID-19 infection [8,9,14]. However, there is currently little published data available to predict how antibody levels against the spike protein will affect clinical outcomes in patients with severe COVID-19 vaccine breakthrough infections. It has yet to be determined which anti-CoV2S antibody level is sufficient for patients with COVID-19 disease to protect against the need for ICU treatment and death. It is thus entirely unclear whether measurement of anti-CoV2S antibody levels in patients with breakthrough infections in the emergency department may aid in risk stratification and clinical decision-making.

In unvaccinated patients, the severity of COVID-19 was shown to be correlated with antibody formation over the course of the disease and neutralization potency is highly protective against fatal outcome [15]. Furthermore, patients’ ability to form high levels of anti-CoV2S antibodies in COVID-19 infection seems to be correlated with improved survival, as measured on the 30th day after ICU admission in unvaccinated critically ill patients in the first pandemic wave in spring 2020 [16]. Similarly, anti-CoV2S IgG levels, as well as IgA antibodies at the time of transfer to the ICU were shown to be protective against death [17]. 

However, data regarding anti-CoV2S IgG antibody status at the time of hospitalization are scarce. We could identify only a single study showing that a positive test for anti-CoV2S IgG antibodies upon hospitalization is a predictor for a reduced risk of death. However, this study included patients from March 2020 to March 2021, and thus does not provide solid information on vaccinated patients and vaccine breakthrough infections, since vaccines only became available towards the end of this period. The fact that a positive anti-CoV2S IgG antibody status appears to be a predictor for improved outcome seems to indicate the importance of patients’ ability to rapidly develop antibodies during infection, as mentioned above. However, the results of this study must be regarded with caution, since antibody testing was only performed based on individual clinical decisions and not in consecutive patients, possibly resulting in selection bias [18]. 

Our data suggest that higher antibody levels upon patients’ presentation in the emergency department, specifically above 600 U/mL, seem to be protective against fatal outcome in the Delta variant vaccine breakthrough infection. We were able to show, in our population of consecutive patients hospitalized with vaccine breakthrough infection, that most patients who survived vaccination breakthrough infection with the Delta variant had an anti-CoV2S antibody level > 600 U/mL, while the majority of hospitalized patients who died or were treated in the ICU showed lower levels. Furthermore, higher levels of anti-CoV2S antibodies remained a predictor for improved outcome even when adjusted for age and number of comorbidities. 

Several other factors are described in the literature that increase the mortality and risk of ICU admission in patients with COVID-19, such as cardiovascular illness, higher age, male gender, smoking, obesity, and kidney diseases [3,19,20]. Thus, clearly, the anti-CoV2S antibody level is merely one of many predictors associated with severity of disease. The fact that neither age nor the number of comorbidities is significantly associated with death in our cohort may be explained by the small sample size, as well as selection bias due to only hospitalized patients being included. The mean age in our study was 68.98 years; most younger patients were probably not hospitalized or did not even present themselves to the emergency department in the first place.

The presented data can be used for further studies to optimize vaccination strategies by using the anti-CoV2S antibody level as a biomarker for protection against death and ICU in patients with COVID-19 disease requiring hospitalization. In addition, this may support the idea of monoclonal antibody therapy as an important tool for the treatment of COVID-19, including breakthrough infections in patients with low antibody levels [21]. Importantly, based on their predictive value, antibody levels upon presentation in an emergency department may, among others, be used as an additional parameter to identify patients at higher risk for severe course and death and may thus facilitate decisions regarding the choice of therapy, especially the decision of whether to start specific antiviral therapy or even help clinicians to decide whether patients need to be hospitalized.

There are several limitations to this retrospective study. We only examined a small sample size of 80 patients. Our findings did not include the duration between the last vaccination and symptom onset nor coinfections that may increase the risk of death [3]. The possible protective value of even higher antibody levels above the detection range used in this study (>2500 U/mL) could not be evaluated in this study. Importantly, data from this study only comprise patients infected with the Delta variant and the associations observed in this study may not extend to breakthrough infections with other variants such as Omicron, which has important immune-escaping properties. Furthermore, T-cell response is an important factor that correlates with protection. We only describe the role of antibodies in mortality and need for intensive care and did not analyze markers of cellular immunity after vaccination. 

## 5. Conclusions

In conclusion, in this retrospective data analysis, we were able to show that almost all patients in our study who died from Delta variant COVID-19 vaccine breakthrough infection had a low antibody level (<600 U/mL) and many of them were even below 200 U/mL. We were also able to show similar results for severely ill patients who had to be treated in the ICU. To determine anti-CoV2S antibody levels sufficient to protect against death and the need for ICU treatment and let antibody levels influence clinical decisions, further studies are needed. There is a significant difference in antibody levels upon presentation between deceased patients and survivors (*p* = 0.00108), and even when adjusted for age and the number of comorbidities, anti-CoV2S antibody levels remain a significant factor protective against death (OR 0.402 for every 1000 U/mL; *p* = 0.018). 

In summary and to the best of our knowledge, this is the first study to indicate that high anti-CoV2S antibody levels upon hospitalization are a significant predictor for improved outcome in COVID-19 vaccine breakthrough infection.

## Figures and Tables

**Table 1:** Hospitalized patients with COVID-19 breakthrough infection.

Variable	n = 80
Gender, N (%)	
Males	75 (93.7)
Females	5 (6.3)
Mean age (±SD)	70 (±15.5)
Duration of hospital stay in days (±SD)	10 (±8)
Comorbidity, N (%)	
BMI > 25 kg/m^2^	33 (41.3)
Arterial hypertension	45 (56.3)
Malignant disease	11 (13.8)
Lung disease	25 (31.3)
Cardiovascular disease	43 (53.8)
Diabetes mellitus	26 (32.5)
Immunosuppression	4 (5)
Presenting symptoms, N (%)	
Cough	43 (53.8)
Fever	40 (50)
Dyspnea	39 (48.8)

Clinical characteristics and demographic data of the hospitalized patients with COVID-19 vaccination breakthrough infection. N = absolute number, SD = standard deviation, n = all hospitalized patients.

## Data Availability

The data presented in this study are available on request from the corresponding author. The data are not publicly available due to privacy considerations.
